# Buyang Tongluo Decoction Mitigates Acute Ischemic Stroke by Suppressing NLRP3/Caspase-1/GSDMD Pathway-Induced Pyroptotic Cell Death

**DOI:** 10.5812/ijpr-171161

**Published:** 2026-06-09

**Authors:** Hai Lin, Yi Liu, Yongjie Gao, Weiyu Xu, Qi Li, Min Li, Kaina Wang

**Affiliations:** 1 Department of Encephalopathy, Xi'an Hospital of Traditional Chinese Medicine, Xi'an, China; 2 Shaanxi University of Chinese Medicine, Xianyang, China

**Keywords:** NLRP3/caspase-1/GSDMD Signaling Pathway, Pyroptosis, Acute Ischemic Stroke, Emergency Database, EMS, Buyang Tongluo Decoction

## Abstract

**Background:**

Acute ischemic stroke (AIS) is associated with neuroinflammation and pyroptotic cell death. The NLRP3/Caspase-1/GSDMD signaling pathway plays an important role in ischemia-induced inflammatory injury. Buyang Tongluo Decoction (BTD), a modified traditional Chinese medicine formulation derived from Buyang Huanwu Decoction (BHD), may exert neuroprotective effects in AIS.

**Objectives:**

This study investigated the neuroprotective effects of BTD in AIS and their association with inhibition of pyroptosis pathways involving NLRP3, Caspase-1, and GSDMD.

**Methods:**

A focal cerebral ischemia–reperfusion model was established using a modified filament occlusion technique. Experimental animals were assigned to four groups: sham controls, untreated model animals, BTD-treated animals, and a positive comparator group receiving BHD. Post-intervention evaluations included neurological assessment using Longa scores, cerebral perfusion analysis by laser speckle imaging, and quantification of ischemic lesions by 2,3,5-triphenyltetrazolium chloride (TTC) staining. Histological examination of brain tissue and neuronal architecture was performed using Nissl and hematoxylin and eosin (H&E) staining, and cellular ultrastructure was assessed using transmission electron microscopy. Protein expression levels of NLRP3, Caspase-1, GSDMD, and HIF-1α were analyzed by Western blotting, and inflammatory cytokine concentrations were determined by enzyme-linked immunosorbent assay. The primary outcomes were neurological score and cerebral infarct volume. Secondary outcomes included cerebral blood flow, histological changes, ultrastructural findings, pyroptosis-related protein expression, and inflammatory cytokine levels.

**Results:**

Compared with the untreated model group, neurological impairment scores were significantly reduced in both the BTD and BHD groups, with BTD showing a greater trend toward neurological recovery. Cerebral perfusion was reduced and infarct volume was increased in untreated model animals, whereas these pathological changes were alleviated by BTD and BHD. BTD treatment was associated with marginally smaller infarct volumes than BHD. Histopathological examination of untreated specimens revealed substantial neuronal injury and disruption of tissue architecture, accompanied by characteristic pyroptotic changes at the ultrastructural level. Both interventions restored morphological and ultrastructural features. In addition, untreated model animals showed increased expression of pyroptosis-related proteins and elevated inflammatory markers, which were significantly suppressed after BTD and BHD administration. Comparative analysis indicated that BTD showed a stronger trend toward modulation of these molecular pathways.

**Conclusions:**

Buyang Tongluo Decoction exerted neuroprotective effects by alleviating neurological dysfunction and reducing cerebral tissue injury. These effects were associated with the suppression of NLRP3/Caspase-1/GSDMD pathway-mediated pyroptosis and inflammatory responses.

## 1. Background

Acute ischemic stroke (AIS) is a common neurological disorder characterized by vascular occlusion or pathological changes in cerebral blood vessels that result in insufficient blood supply to brain tissue. This condition triggers oxygen deprivation and subsequent neuronal cell death, ultimately causing sudden disruption of cerebral circulation. In traditional Chinese medicine (TCM), AIS falls under the category of “Zhongfeng” (stroke), which is characterized by rapid onset, rapid progression, high incidence, and high recurrence (1, 2). Acute ischemic stroke poses a substantial threat to human health and is associated with increasing prevalence and mortality. Globally, approximately 7.8 million new cases are diagnosed annually, with an estimated 3.59 million related deaths. With progressive population aging worldwide, the incidence of this cerebrovascular disorder continues to increase. In China, AIS has become the leading cause of physical disability among adults, representing a major public health concern and generating substantial socioeconomic pressure on affected families and communities (3). Therefore, strengthening the clinical management of AIS remains an urgent priority.

The pathological progression of AIS involves marked neuroinflammation, and pyroptosis has emerged as a key inflammatory form of cell death linked to caspase-dependent pathways (4). Pyroptosis induces inflammatory cell death and is primarily characterized by cell swelling and lysis, DNA damage, chromatin condensation, and membrane rupture (5). Continued investigation of the molecular mechanisms of pyroptosis has revealed the pivotal regulatory role of inflammasomes, which broadly influence various cell death modalities, including pyroptosis, ferroptosis, and apoptosis. These findings underscore the complex interplay between inflammatory mechanisms and neuronal damage in ischemic stroke.

The NLRP3 inflammasome is an intracellular pattern-recognition receptor complex that comprises multiple protein components, including NLRP3 (NOD-like receptor family pyrin domain-containing protein 3), ASC (apoptosis-associated speck-like protein containing a caspase recruitment domain), and pro-Caspase-1 (6). Upon detection of danger-associated molecular patterns, NLRP3 undergoes structural modification and forms oligomeric structures. This process exposes the PYD domain at the N-terminus, enabling interaction with the corresponding PYD domain of ASC through homotypic binding. These molecular interactions facilitate the formation of ASC specks, which have prion-like aggregation properties. Subsequently, the CARD domain of ASC interacts with pro-Caspase-1, leading to complete assembly of the NLRP3 inflammasome. The fully assembled complex then induces self-cleavage and activation of pro-Caspase-1 through mechanisms involving close molecular proximity (7, 8).

The molecular mechanism of pyroptosis is predominantly mediated by Caspase-1 activation. Caspase-1 cleaves Gasdermin D (GSDMD), generating active fragments. The N-terminal fragments of GSDMD subsequently interact with membrane phospholipids and form transmembrane pores that compromise cellular osmotic homeostasis. This membrane disruption enables the extracellular release of processed inflammatory cytokines, particularly IL-1β and IL-18 (9, 10). This process further amplifies inflammatory responses and ultimately triggers pyroptotic cell death. Emerging research indicates that Caspase-1 expression and activity are significantly elevated in both neurons and microglial cells after AIS, highlighting the critical involvement of this canonical pyroptotic pathway in cerebral infarction pathology (11, 12). Therefore, inhibiting pyroptosis has important clinical significance for improving AIS outcomes.

Modern medicine has made significant advances in elucidating the pathophysiological mechanisms of AIS. Current clinical therapies for ischemic stroke, including thrombolysis, anticoagulation, antiplatelet therapy, and strategies to improve cerebral circulation, have yielded moderate therapeutic effects by targeting different pathological stages of the disease (13-15). However, the overall therapeutic effect in AIS remains unsatisfactory, and most patients remain at substantial risk of residual neurological dysfunction, which severely affects quality of life. The number of studies investigating TCM interventions for AIS has increased annually, highlighting the increasingly prominent advantages of TCM in the treatment of AIS (16). Pyroptotic cell death regulated through the NLRP3/Caspase-1/GSDMD signaling pathway plays a critical role in cerebral injury after AIS (17).

Buyang Tongluo Decoction (BTD) is a TCM prescription formulated by modifying the classic Buyang Huanwu Decoction (BHD) based on practical application scenarios. Buyang Huanwu Decoction is a long-established therapeutic preparation for stroke-related diseases and is derived from the Qing Dynasty medical text “Yilin Gaicuo.” Contemporary pharmacological research has demonstrated that BHD is effective in managing ischemic stroke by suppressing inflammatory processes (18). As a long-established TCM formula, BHD has well-documented anti-inflammatory and cell-protective functions (18-20). Including BHD as a positive comparator enables benchmarking of the efficacy of BTD against a clinically relevant TCM standard.

## 2. Objectives

Based on existing evidence, this experiment further verified the therapeutic effect of BTD in AIS. Specifically, the study examined whether BTD protects neural tissue against AIS by modulating pyroptosis through the NLRP3/Caspase-1/GSDMD signaling cascade. These findings provide theoretical and practical support for further preclinical investigation of BTD in AIS management.

## 3. Methods

### 3.1. Medicinal Materials and Preparation

The formulations of BTD and BHD are presented in Table S1 in the Supplementary File. All raw herbal materials were obtained from the Traditional Chinese Medicine Pharmacy at Xi'an Hospital of Traditional Chinese Medicine and were identified as meeting the legal standards for the corresponding varieties in the Pharmacopoeia of the People's Republic of China (2020 edition, Volume I). Each batch of herbal decoction was standardized by yield and concentration.

For preparation, BTD and BHD were soaked in 500 mL of purified water (grade III) for 30 minutes. Additional purified water (grade III) was added at a weight-to-volume ratio of 1:8. The medicinal herbs and water were placed into a decoction machine and boiled 3 times for 1.5 hours, 1 hour, and 45 minutes, respectively. After the decoction solutions were combined, they were concentrated and precipitated overnight with ethanol. After filtration and ethanol distillation, the preparations were concentrated and stored in 500-mL wide-mouth reagent bottles at 4°C in a refrigerator.

Methodological validation of astragaloside IV content determination in the BTD compound preparation was completed. The method met the requirements for specificity, linearity, repeatability, accuracy, intermediate precision, and robustness. The average astragaloside IV content in BTD was 0.68 mg/g. Batch consistency was controlled based on astragaloside IV content.

The dosage was calculated using the body surface area method for equivalent dose conversion between humans and rats. A standard adult body weight of 70 kg and a rat body weight of 200 - 250 g were used as baselines. A human-to-rat dose conversion factor of 6.25 was applied in combination with the clinical adult dosage. The dosage volume for the BTD group was 0.9 mL/250 g per administration, and that for the BHD group was 0.675 mL/250 g per administration.

### 3.2. Preparation of the Acute Ischemic Stroke Model

A rat model of focal cerebral ischemia was induced using an adapted filament occlusion technique targeting the right hemisphere. Animals were anesthetized by intraperitoneal injection of ketamine (70 mg/kg) combined with xylazine (5 mg/kg) and then placed in the dorsal recumbent position on a thermoregulated heating platform to maintain core body temperature at 37°C throughout the operation. Postoperative analgesia was provided by administering buprenorphine (0.05 mg/kg, subcutaneously) every 12 hours for the first 48 hours after surgery.

After antiseptic preparation of the cervical region, a 2-cm midline skin incision was made. Through blunt dissection, the submandibular gland fascia was separated to expose the right sternocleidomastoid and sternohyoid muscles. The intermuscular space was carefully dissected to visualize the carotid sheath. Under microscopic guidance, the right common carotid artery (CCA) was meticulously isolated while preserving the adjacent vagus nerve. Subsequent dissection exposed the internal carotid artery (ICA) and external carotid artery (ECA). Temporary vascular occlusion was achieved by clamping the ICA, while simultaneous occlusion was applied to both the ECA and the proximal segment of the CCA.

The CCA was carefully ligated, and a small V-shaped incision was made in its wall. A suture thread was then introduced into the ICA through the CCA and carefully advanced toward the skull until it reached the origin of the middle cerebral artery. The optimal insertion distance was approximately 18 - 20 mm from the bifurcation of the internal and external carotid arteries, and advancement was stopped when mild resistance was encountered. The suture plug was ligated and fixed, the excess suture plug was removed, streptomycin was sprinkled on the surgical area, and the muscle and skin were sutured layer by layer.

Throughout the procedure, a laser speckle imaging system continuously monitored blood flow changes in the affected brain region, and a substantial reduction in cerebral perfusion was used as the key indicator of successful model establishment. At 1.5 hours after surgery, the suture plug was removed for reperfusion, and subsequent experiments were conducted 24 hours after reperfusion.

The following exclusion criteria were applied: 1) subarachnoid hemorrhage confirmed by postmortem inspection; 2) Longa score < 1 at 24 hours after modeling; and 3) death during surgery. All animals were assessed for these criteria immediately after surgery and at 24 hours. In the model group, 2 animals met criterion 1 and were excluded before any further analysis; they were replaced by new animals that underwent the same modeling procedure to maintain n = 12 for neurological scoring.

Humane endpoints were predefined as follows: 1) body weight loss exceeding 20% of baseline; 2) inability to access food or water; and 3) a moribund state characterized by lethargy, hypothermia, and labored breathing. Animals reaching any of these endpoints were immediately euthanized by pentobarbital sodium overdose (200 mg/kg, intraperitoneally).

### 3.3. Experimental Animals and Grouping

Male Sprague-Dawley rats (6 - 8 weeks old; body weight, 200 - 250 g) certified as specific pathogen-free were obtained from Shaanxi Pharmaceutical Medical Biotechnology Company. The animals were housed under controlled environmental conditions (22 ± 2°C, 12-hour light/dark cycle), with free access to food and water during a 7-day adaptation period.

Allocation concealment was achieved using sequentially numbered, opaque, sealed envelopes prepared by an independent researcher who was not involved in animal surgery or outcome assessment. The randomization sequence was generated using SPSS version 26.0 (IBM Corporation, USA) and was kept concealed until group assignment. Animal welfare monitoring was performed twice daily (8:00 AM and 8:00 PM) throughout the experimental period. Body weight, food and water intake, general appearance, and neurological status were observed.

The experimental design included the following treatment conditions (n = 12):

1) Sham group: Rats underwent skin incision and suture insertion without vessel ligation, mirroring all other operative steps performed in the model group. Oral administration of normal saline was initiated on the second day after surgery, twice daily for 2 consecutive weeks.

2) Model group: A focal cerebral ischemia-reperfusion model was prepared using an improved intraluminal suture method. Starting on the second day after surgery, normal saline was administered orally twice daily for 2 consecutive weeks.

3) BHD treatment group: Oral administration of BHD was initiated on the second day after surgery, 3 times daily for 2 consecutive weeks at a dosage volume of 0.675 mL/250 g/time.

4) BTD treatment group: Oral administration of BTD was initiated on the second day after surgery, twice daily for 2 consecutive weeks at a dosage volume of 0.9 mL/250 g/time.

The entire outcome evaluation process was conducted in a blinded manner. For endpoint-specific assays, including cerebral blood flow, infarct volume, Western blotting, and enzyme-linked immunosorbent assay (ELISA), animals were randomly subsampled from each group using a separate computer-generated randomization list to ensure that the animals selected for different assays were representative of the whole cohort. Staff responsible for neurological function scoring, cerebral blood flow measurement, infarct volume analysis, histological evaluation, transmission electron microscopy observation, Western blot quantification, and ELISA detection were unaware of the group assignments.

For neurological function scoring, 12 animals from each group were tested. For cerebral blood flow measurement, 5 animals were randomly selected from each group of 12. For infarct volume, Western blotting, and ELISA, 3 animals were randomly selected from each group of 12.

The research procedures were approved by the Experimental Animal Ethics Committee of Shaanxi University of Chinese Medicine (Approval No. SNCMDL20250903003), and all animal experiments were conducted in full compliance with China's established standards for laboratory animal welfare and use. This investigation adhered to the updated ARRIVE 2.0 experimental guidelines (21).

### 3.4. Evaluation of Neurological Dysfunction Using the Longa Score

The neurological status of all experimental rodents was assessed using the Longa scale at 2 time points: 24 hours after model establishment and 14 days after treatment initiation. Higher scores indicated greater neurological dysfunction. The scoring system was as follows: 0, normal neurological function; 1, observable flexion and adduction of the contralateral forelimb when the tail was elevated; 2, circular movement toward the affected side during locomotion; 3, loss of balance toward the impaired side while moving or standing; and 4, complete absence of voluntary movement accompanied by impaired consciousness.

Two animals from the model cohort were eliminated from the study because of subarachnoid hemorrhage. These animals were replaced with additional rats to maintain n = 12 per group for neurological scoring and mortality-sensitive outcomes.

### 3.5. Detection of Cerebral Cortical Blood Flow

The rats were anesthetized using ketamine (70 mg/kg) and xylazine (5 mg/kg) before being secured on the surgical platform. A cutaneous incision was made over the cranial region of interest to expose the underlying bone structure. The laser-based microcirculation monitoring device (Moor Instruments, UK) was carefully positioned on the exposed cranial surface, with deliberate avoidance of the midline suture, to measure regional cerebral cortical perfusion.

### 3.6. Quantification of Cerebral Infarct Volume by TTC Staining

At the end of the experimental procedures, the rats underwent transcardiac perfusion with chilled phosphate-buffered saline (PBS). The animals were then euthanized by decapitation, and their brains were promptly excised and placed in a -20°C environment for 30 minutes. The cryopreserved cerebral tissue was subsequently removed and precisely divided into 5 equidistant coronal sections, each 2 mm thick, while maintained on ice. All sections were immersed in 2% 2,3,5-triphenyltetrazolium chloride (TTC) solution (Sigma-Aldrich, USA) and incubated in the dark at room temperature for 20 minutes. After staining, the tissue samples were preserved in 4% paraformaldehyde and systematically arranged for photographic documentation. Viable neural tissue appeared red, whereas ischemic regions remained unstained and appeared pale white. Cerebral infarction was quantified using digital image analysis with ImageJ software (NIH, USA), and the infarct percentage was determined using the following equation: Percentage of cerebral infarction = (Aggregate volume of infarct region across all sections / Total sectional brain volume) × 100%.

### 3.7. Nissl Staining

At the end of the experimental procedures, the rodents underwent transcardiac perfusion with chilled PBS. The brain was then extracted, with a specific focus on obtaining ischemic hippocampal regions. Tissue blocks were fixed in 4% paraformaldehyde phosphate buffer for 15 minutes, followed by preparation of 10-μm paraffin sections through dehydration, clearing, and wax immersion. The prepared sections were first washed under running water and then stained with Nissl staining reagent (Solarbio, China) for 15 minutes. After staining, the sections were gently rinsed with distilled water. Rapid dehydration was then performed using ascending concentrations of absolute ethanol, followed by xylene clearing for 5 minutes. The sections were sealed with neutral gum and observed under an optical microscope (Olympus, Tokyo, Japan) for image acquisition and analysis.

### 3.8. Hematoxylin and Eosin Staining

Paraffin-embedded tissue samples were first dewaxed using Eco-Friendly Dewaxing Agents I and II, with 20 minutes of incubation in each solution to ensure complete paraffin removal. The samples were then progressively rehydrated through an ethanol gradient consisting of absolute ethanol I and absolute ethanol II, each for 5 minutes, and 75% ethanol for 5 minutes, followed by extensive washing under running tap water.

After hydration, the sections were treated with high-definition staining pretreatment solution for 60 seconds and then immersed in hematoxylin dye for 3 - 5 minutes. After rinsing with tap water, the sections were differentiated with differentiation solution, rinsed again with tap water, blued with blue solution, and rinsed under running water. The sections were then briefly dehydrated in 95% ethanol for 1 minute and counterstained with eosin for 15 seconds. Final dehydration was performed using absolute ethanol twice for 2 minutes each, and the sections were cleared in n-butanol twice for 2 minutes each to enhance transparency. The tissue samples then underwent sequential immersion in ethanol solutions of increasing concentrations (70%, 80%, 90%, and 100%, each for 15 minutes), followed by 2 xylene treatments for 2 minutes each. Finally, the sections were sealed with neutral gum and examined under an optical microscope (Olympus, Tokyo, Japan) for image acquisition and analysis.

### 3.9. Transmission Electron Microscopy

Fresh infarcted brain tissue was immediately dissected into 1-mm^3^ pieces after harvest and fixed in precooled 2.5% glutaraldehyde fixative at 4°C for 4 hours. After 3 rinses with 0.1 M PBS, the tissue pieces were fixed in 1% osmium tetroxide solution (Sigma-Aldrich, USA) in the dark at room temperature for 2 hours. Subsequent processing involved sequential dehydration through increasing ethanol concentrations (50%, 70%, 80%, 90%, and absolute ethanol, each for 15 minutes), followed by propylene oxide treatment, progressive epoxy resin infiltration, and final embedding polymerization. Ultrathin sections measuring 70 nm were prepared using an ultramicrotome. These sections underwent double staining with uranyl acetate in ethanol and lead citrate solution before examination and documentation using a transmission electron microscope (Hitachi High-Tech, Japan).

### 3.10. Western Blotting

Brain specimens were carefully weighed and mechanically disrupted in ice-cold RIPA buffer containing 1% protease inhibitor and 2% phosphatase inhibitor at a 1:10 tissue-to-buffer ratio until complete dissolution was achieved. After homogenization, the mixture was centrifuged at 4°C and 12,000 rpm for 10 minutes, and the resulting supernatant was collected as the protein extract. Protein quantification was performed using a BCA assay kit (Beyotime, China), after which samples were heat-denatured at 100°C for 10 minutes for subsequent analysis.

The prepared protein samples were separated by SDS-PAGE and then transferred onto PVDF membranes (Merck Millipore, USA). Membranes were blocked with 5% nonfat milk at ambient temperature for 60 minutes. Primary antibodies against NLRP3 (BosterBio, China), ASC (Bioss, China), pro-Caspase-1 (Abcam, USA), cleaved Caspase-1 (Affinity Bioscience, USA), GSDMD (Biodragon, China), N-GSDMD (HuaBio, China), and HIF-1α (Bioss, China) were added, and the membranes were incubated overnight at 4°C. Secondary antibodies were incubated with the samples at ambient temperature for 60 minutes. Chemiluminescent detection was then performed using immunoblotting reagents to visualize protein bands. The resulting bands were captured digitally and subjected to quantitative analysis using ImageJ software (NIH, USA), with band intensity measurements providing semiquantitative data.

### 3.11. Enzyme-Linked Immunosorbent Assay

The concentrations of inflammatory mediators, including IL-1β, tumor necrosis factor-alpha (TNF-α), IL-18, and HIF-1α, were quantified using commercial ELISA kits (Beyotime, China) according to the manufacturer's protocols. The assay procedure involved processing brain tissue homogenate supernatants and serum samples through sequential steps, including sample application, incubation, plate washing, reagent addition, chromogenic reaction, and termination. Optical density measurements were recorded at 450 nm.

### 3.12. Statistical Analysis

Statistical analyses were conducted using GraphPad Prism software (version 10.1.2), and results are expressed as mean ± standard deviation (mean ± SD). Before one-way analysis of variance (ANOVA) was used, the normal distribution of the data was verified using the Shapiro-Wilk test, and variance equality was examined using Levene's test. The dataset satisfied both normality and variance homogeneity prerequisites. For repeated measures, including neurological scores at 24 hours and 14 days, 2-way repeated-measures ANOVA was applied, with group and time as factors. For intergroup mean comparisons, ANOVA was used, supplemented by Dunnett post hoc analysis for specific group contrasts. Statistical significance was defined as P < 0.05.

The primary outcomes were neurological function, assessed using the Longa score, and cerebral infarct volume, assessed using TTC staining. Secondary outcomes included cerebral blood flow, histological changes using Nissl and H&E staining, ultrastructural findings using transmission electron microscopy, pyroptosis-related protein expression using Western blotting, and inflammatory cytokine levels using ELISA.

## 4. Results

### 4.1. BTD Improves Neurological Deficits

Neurological assessments conducted 24 hours after modeling showed markedly elevated scores in the model cohort, BTD intervention group, and BHD treatment group compared with the sham-operated controls (P < 0.001), with no significant differences among these 3 experimental groups (Figure 1A). These results indicated successful model establishment and severe neurological impairment in the model group, because drug administration did not begin until 24 hours after modeling. After 2 weeks of treatment, the model group maintained persistently high neurological scores, whereas both the BTD and BHD groups showed substantial score reductions (P < 0.01). These findings suggest that both treatment regimens significantly improved neurological deficits after cerebral ischemia, with the BTD group showing a slightly stronger trend toward improvement than the BHD group (Figure 1B).

**Figure 1.**
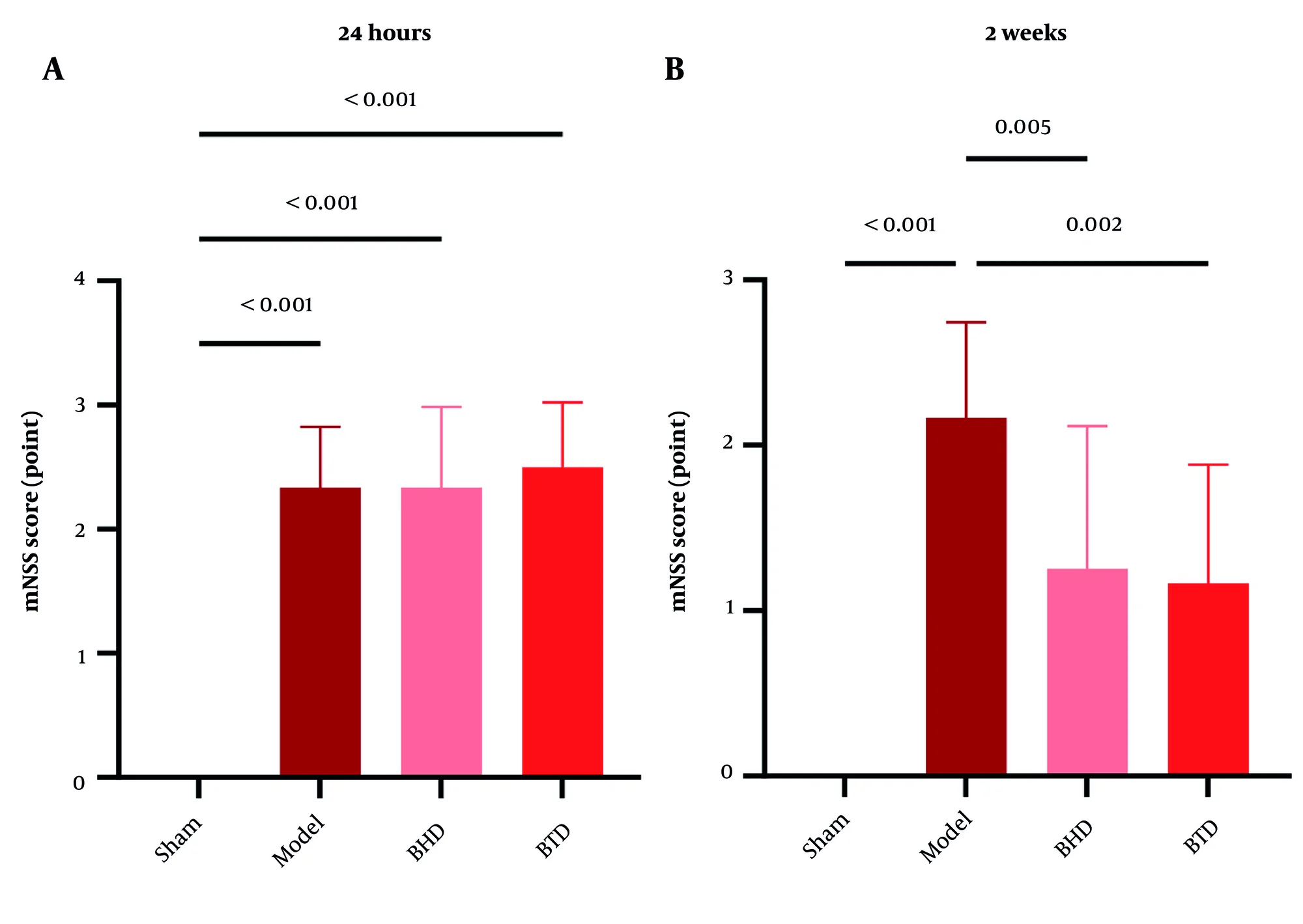
BTD improved neurological deficits in rats with AIS. (A) Neurological scores assessed 24 hours after model establishment, before administration. (B) Neurological scores assessed 2 weeks after administration. Data are presented as mean ± SD (n = 12). Statistical significance was defined as P < 0.05. BTD, Buyang Tongluo Decoction; BHD, Buyang Huanwu Decoction.

### 4.2. BTD Improves Cerebral Blood Flow

Laser speckle imaging showed a marked reduction in cortical perfusion in the surgical hemisphere of rodents in the model group subjected to cerebral ischemia-reperfusion injury, validating successful model establishment. Animals receiving BTD or BHD showed partial restoration of cerebral perfusion after 2 weeks of treatment, indicating that both herbal formulations may promote blood flow restoration in ischemic areas by enhancing cerebral microcirculation or alleviating vascular constriction, thereby creating favorable conditions for subsequent recovery of neural function (Figure 2).

**Figure 2.**
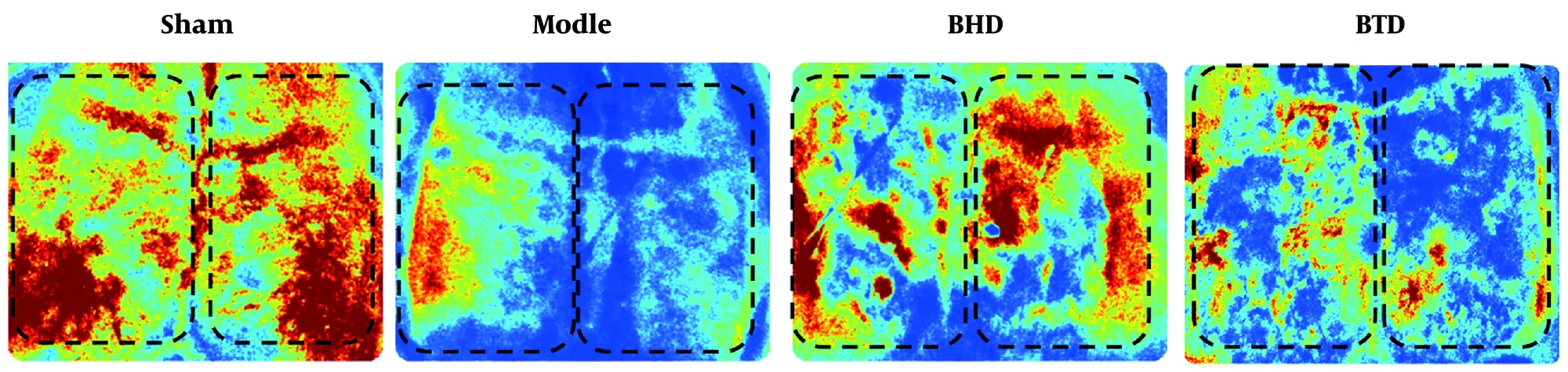
BTD improved cerebral blood flow in rats with AIS. Representative laser speckle images show cerebral blood flow on the surgical side in each group. BTD, Buyang Tongluo Decoction; BHD, Buyang Huanwu Decoction.

### 4.3. BTD Reduces Cerebral Infarct Volume

TTC staining showed markedly larger infarct volumes in the model group than in the sham group (P < 0.001). Administration of either BTD or BHD led to substantial decreases in infarct size (P < 0.05), confirming the neuroprotective properties of both herbal formulations against ischemic brain damage. Comparative analysis showed marginally better outcomes with BTD than with BHD in terms of infarct reduction (Figure 3).

**Figure 3.**
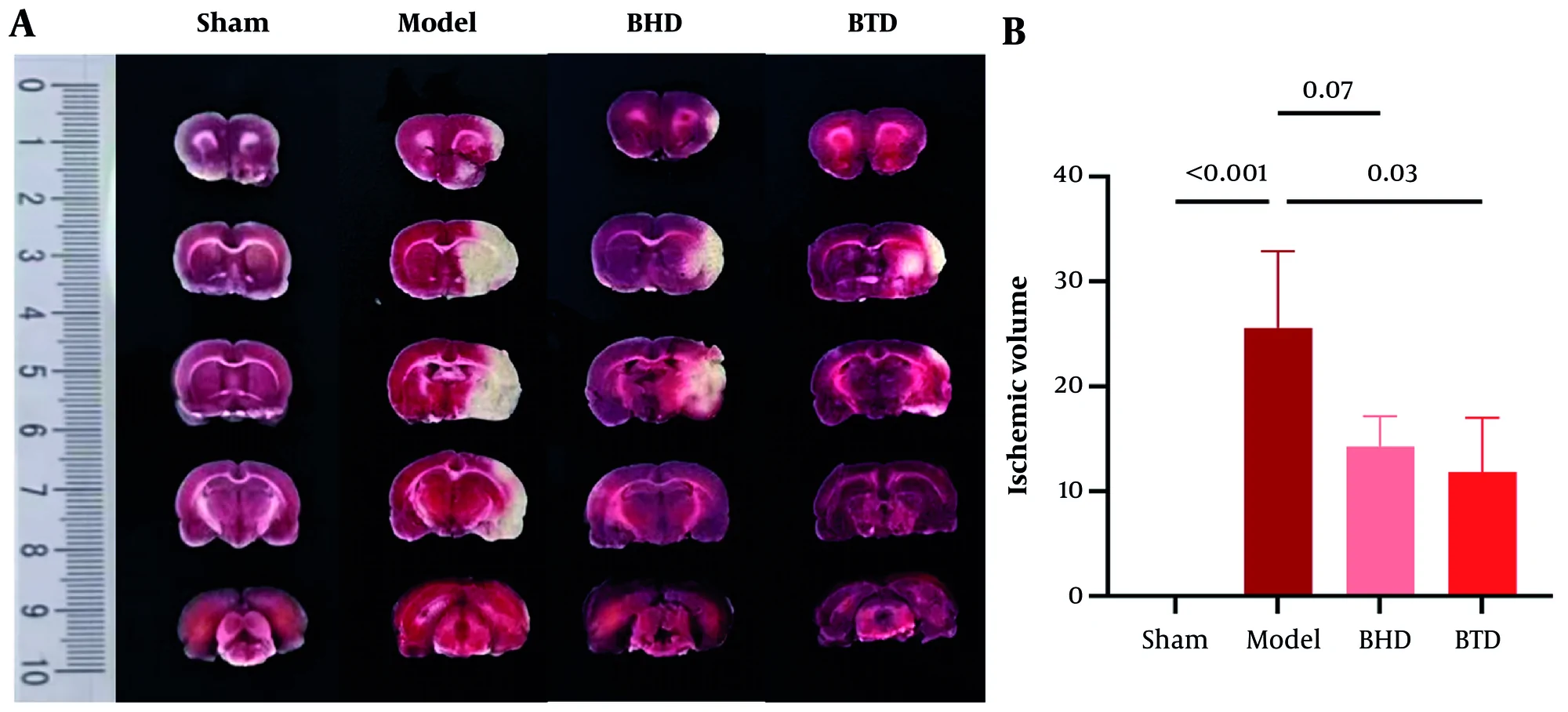
BTD reduced cerebral infarct volume in rats with AIS. (A) Representative TTC-stained coronal brain sections show the infarct area (white) in each group. (B) Quantitative analysis of cerebral infarct volume. Data are presented as mean ± SD (n = 3). Statistical significance was defined as P < 0.05. BTD, Buyang Tongluo Decoction; BHD, Buyang Huanwu Decoction.

### 4.4. BTD Alleviates Neuronal Damage in Brain Tissue

Histological examination using Nissl staining showed distinct morphological differences among the experimental groups. The sham-operated control group displayed neurons with well-defined cellular contours, intact neurites, and tight organization. These cells showed abundant and evenly distributed Nissl substance with intense basophilic staining. In contrast, the model group showed significant neuronal degeneration characterized by shrunken somata, disrupted processes, disorganized cellular architecture, and substantially diminished Nissl bodies. Both the BTD and BHD intervention groups showed notable neuroprotective effects compared with the model group, as evidenced by increased neuronal density, improved somatic morphology, and partial recovery of Nissl body staining intensity. These findings indicate that therapeutic administration of either BTD or BHD effectively ameliorated neuronal injury (Figure 4).

**Figure 4.**
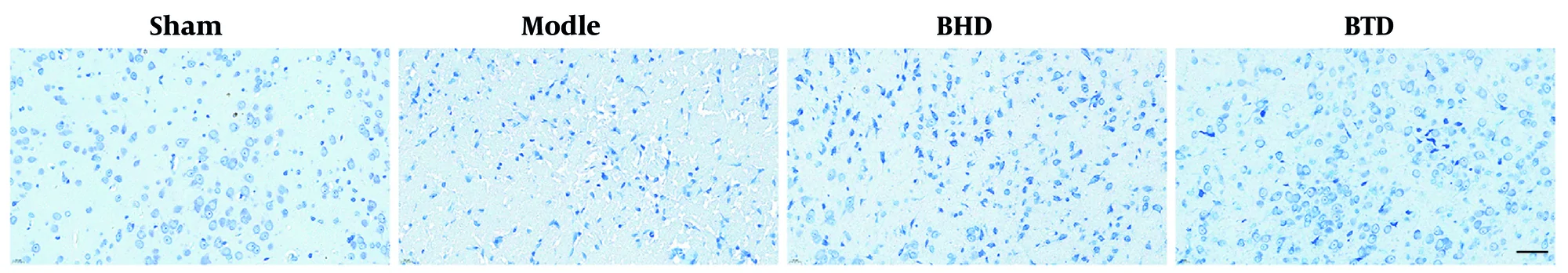
BTD alleviated neuronal damage in AIS brain tissue. Representative images of Nissl staining show neuronal morphology in the hippocampal region (scale bar = 50 μm). BTD, Buyang Tongluo Decoction; BHD, Buyang Huanwu Decoction.

### 4.5. BTD Improves the Morphology of Brain Tissue Cells

Histopathological examination using H&E staining showed distinct morphological differences among the experimental groups. In control specimens, cerebral tissue displayed normal cytoarchitecture characterized by tightly packed neurons of uniform size and shape. These cells had prominent, centrally positioned nuclei with clear nuclear membranes and evenly distributed chromatin. In the model group, many neurons were sparsely distributed, with irregular outlines and cellular atrophy. Interstitial gaps and vacuoles resulting from cellular atrophy were visible around the cells, and nuclear chromatin was dense with markedly darker staining. Therapeutic intervention with BTD and BHD resulted in substantial histological improvements, with the treated groups showing preserved neuronal morphology, reduced cytoplasmic shrinkage, and marked mitigation of extracellular space abnormalities compared with untreated model specimens. Nuclear staining intensity was markedly decreased. These results suggest that both BTD and BHD treatment can effectively improve the morphological structure of brain tissue cells damaged by AIS (Figure 5).

**Figure 5.**
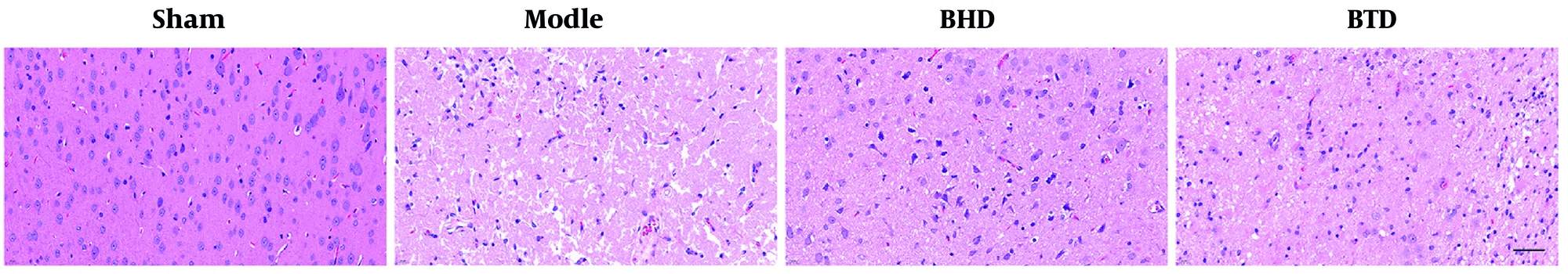
BTD improved the morphological structure of brain tissue cells. Representative images of H&E staining show cellular morphology in the infarcted cortex (scale bar = 50 μm). BTD, Buyang Tongluo Decoction; BHD, Buyang Huanwu Decoction.

### 4.6. BTD Downregulates the Expression of Pyroptosis-Related Proteins

Protein expression analysis showed markedly elevated expression of NLRP3, ASC, pro-Caspase-1, cleaved Caspase-1, N-GSDMD, and HIF-1α in the model group (P < 0.05), indicating activation of pyroptosis after cerebral ischemic injury. Administration of BTD and BHD resulted in substantial suppression of these protein markers, demonstrating that both therapeutic agents effectively attenuated expression of the NLRP3/Caspase-1/GSDMD pathway (Figure 6).

**Figure 6.**
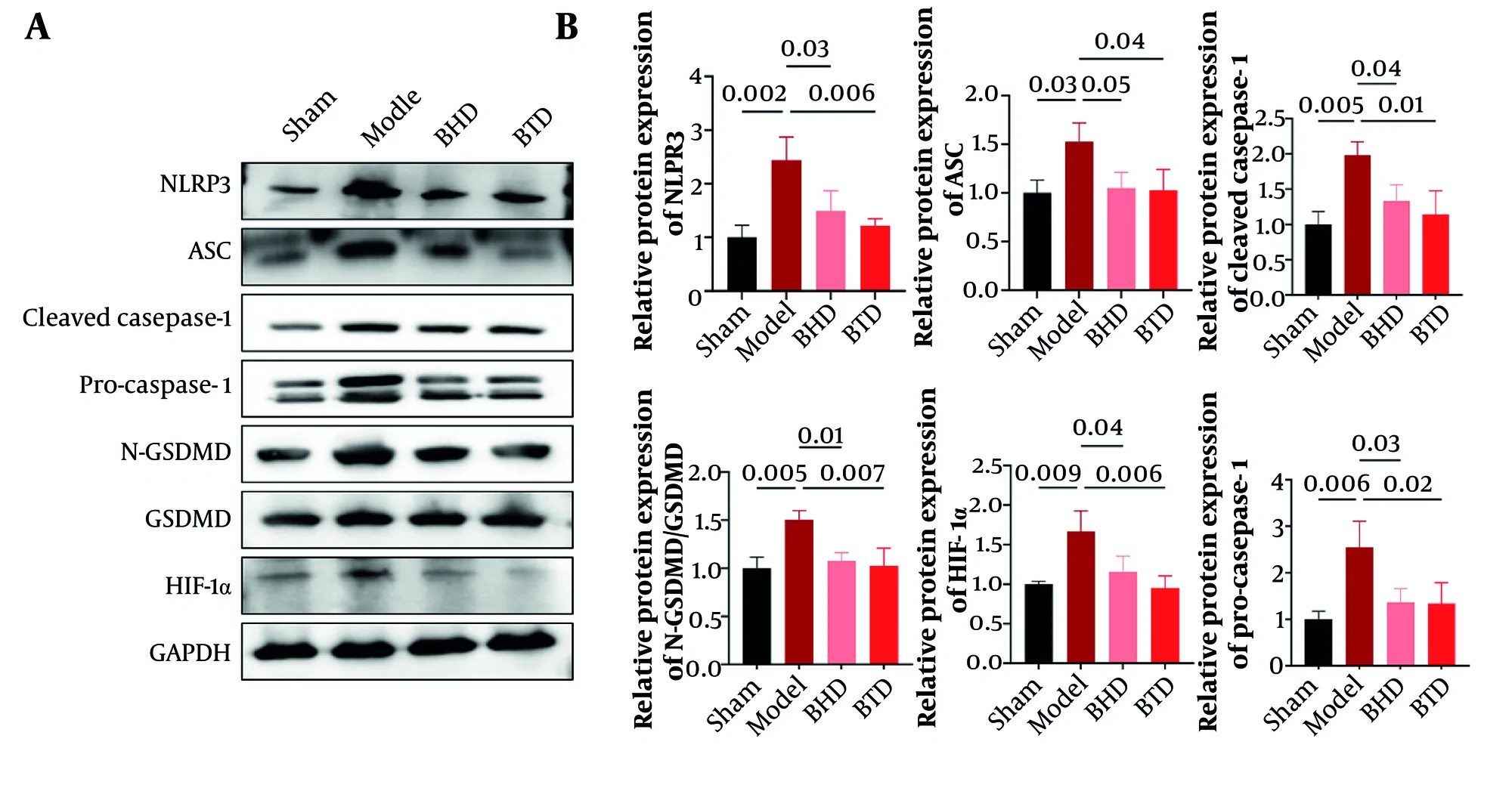
BTD downregulated the expression of pyroptosis-related proteins in brain tissue. (A) Representative Western blot bands show the expression levels of NLRP3, ASC, pro-Caspase-1, cleaved Caspase-1, GSDMD, N-GSDMD, and HIF-1α. (B) Quantitative analysis of protein expression levels normalized to GAPDH. Data are presented as mean ± SD (n = 3). Statistical significance was defined as P < 0.05. BTD, Buyang Tongluo Decoction; BHD, Buyang Huanwu Decoction.

### 4.7. Ultrastructural Analysis

Transmission electron microscopy showed that neurons in the model group displayed distinct pyroptotic features, including perforated plasma membranes, disrupted nuclear membranes, and enlarged mitochondria. After treatment with BTD and BHD, cell membrane integrity improved and mitochondrial structure returned toward normal, confirming at the ultrastructural level that both agents can inhibit pyroptosis and preserve cell membrane structure and mitochondrial function (Figure 7).

**Figure 7.**
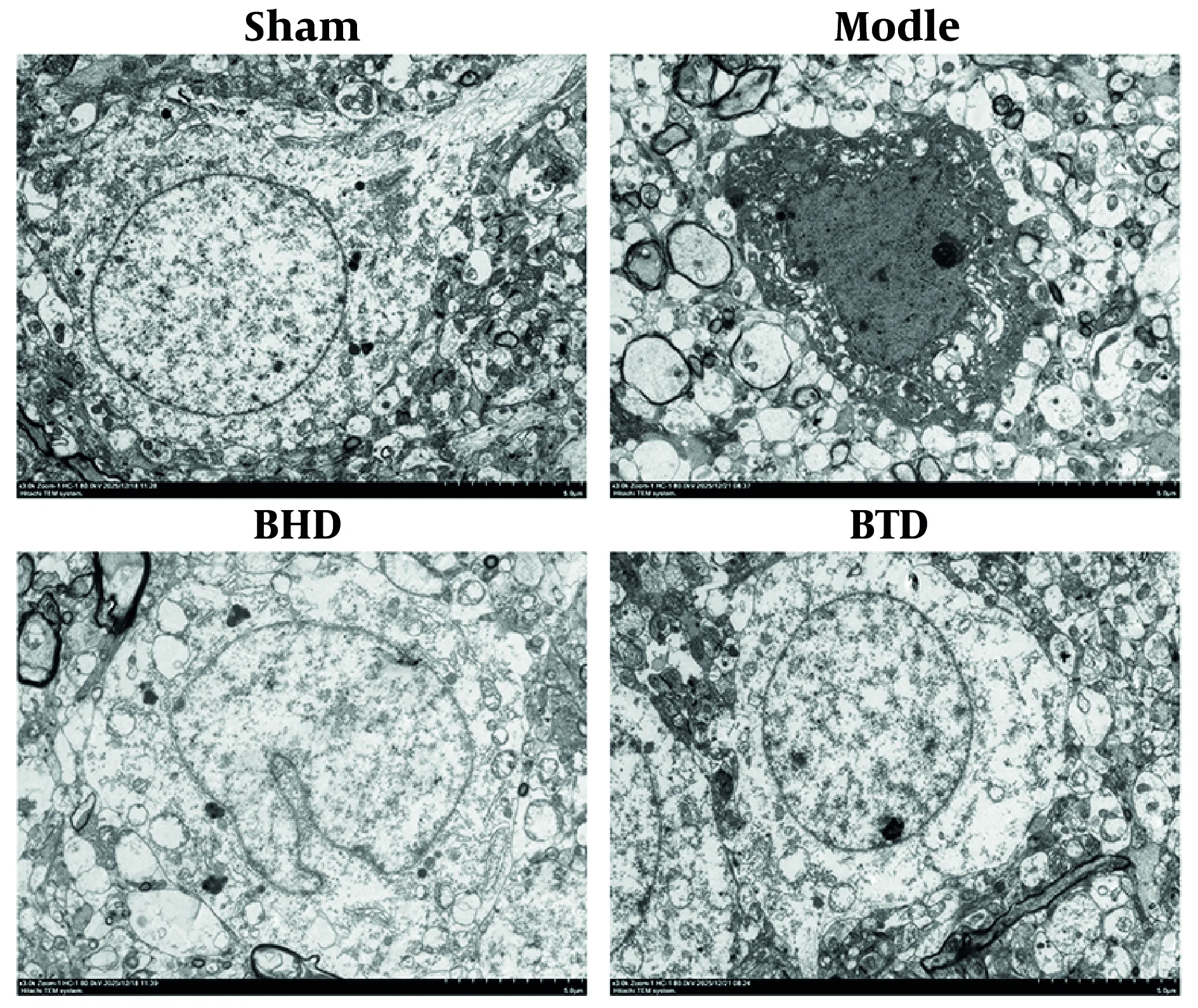
BTD ameliorated neuronal ultrastructural damage. Representative transmission electron microscopy images show neuronal ultrastructure in infarcted brain tissue (scale bar = 5 μm). BTD, Buyang Tongluo Decoction; BHD, Buyang Huanwu Decoction.

### 4.8. BTD Reduces Inflammatory Cytokine Levels

ELISA results showed markedly increased concentrations of IL-1β, TNF-α, IL-18, and HIF-1α in both serum and cerebral tissues in the model group. Administration of BTD and BHD led to differential reductions in these proinflammatory mediators, providing additional evidence that these herbal formulations mitigate postischemic inflammation by suppressing pyroptotic pathways and reducing the secretion of inflammatory factors (Figure 8).

**Figure 8.**
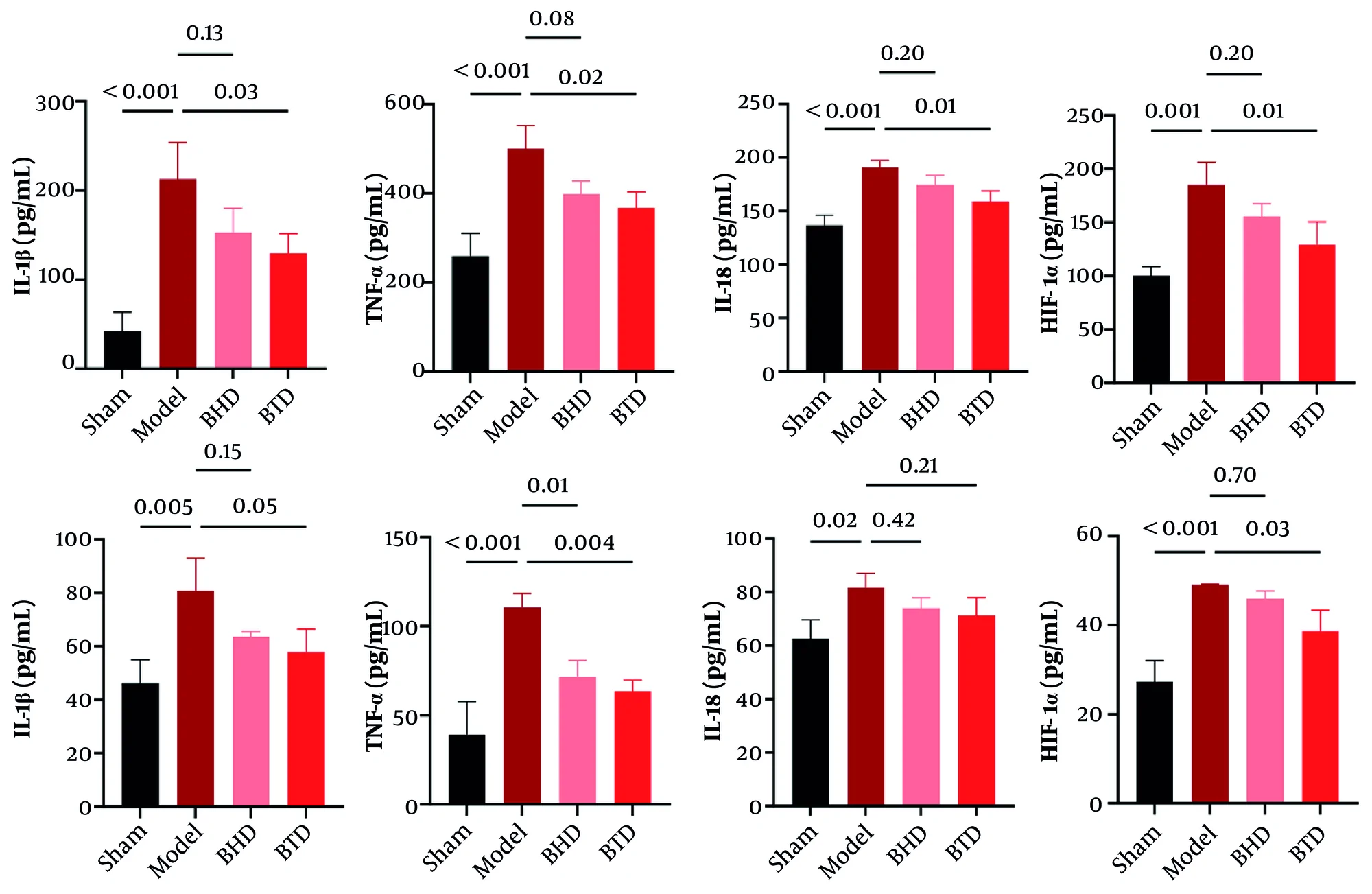
BTD reduced inflammatory cytokine levels in serum and brain tissue. (A) Levels of IL-1β, TNF-α, IL-18, and HIF-1α in serum and (B) brain tissue homogenates measured by ELISA. Data are presented as mean ± SD (n = 3). Statistical significance was defined as P < 0.05. BTD, Buyang Tongluo Decoction; BHD, Buyang Huanwu Decoction.

## 5. Discussion

This study used a rat model of AIS to comprehensively evaluate the neuroprotective effects of BTD against cerebral ischemia and to explore its molecular mechanisms. The findings demonstrated that BTD administration markedly improved neurological function, reduced ischemic brain tissue volume, alleviated ultrastructural neuronal injury, and suppressed the protein levels of NLRP3 inflammasome components, including ASC, pro-Caspase-1, cleaved Caspase-1, and N-GSDMD, as well as those of inflammatory cytokines such as IL-1β, TNF-α, and IL-18, and hypoxia-inducible factor HIF-1α. The observed neuroprotection appears to be primarily mediated by suppression of pyroptotic cell death through modulation of the NLRP3/Caspase-1/GSDMD signaling cascade. It should be noted that TCM formulations typically have complex pharmacological profiles involving multiple bioactive constituents and diverse molecular targets. The anti-pyroptosis mechanism identified in this investigation likely represents one important component of the broader therapeutic actions of BTD.

Inflammation and subsequent tissue damage in AIS are strongly influenced by pyroptosis, a form of programmed cell death (22). Studies have shown that TCM formulations such as Taohong Siwu Decoction, Naoxinqing Capsules, and BHD can exert neuroprotective effects by inhibiting pyroptosis, thereby treating or improving cerebral infarction (22, 23). The experimental data from this investigation showed marked increases in NLRP3, ASC, cleaved Caspase-1, and N-GSDMD protein levels in the model group, along with increased secretion of the proinflammatory mediators IL-1β and IL-18. Ultrastructural examination by transmission electron microscopy identified characteristic pyroptosis-associated morphological changes in neuronal cells, including plasma membrane perforation and mitochondrial distension. These findings provide evidence that acute cerebral ischemia activates the NLRP3/Caspase-1/GSDMD-mediated pyroptosis cascade in rodent brain tissue.

Administration of BTD resulted in significant modulation of these molecular markers. Analyses of biochemical and structural indicators showed notable improvements, strongly suggesting that BTD suppresses pyroptotic processes by interfering with NLRP3 inflammasome assembly, preventing Caspase-1 activation, and inhibiting GSDMD cleavage. Collectively, these mechanisms contribute to reduced inflammatory responses and prevention of neuronal cell death. These results provide pharmacological and molecular support for the therapeutic application of BTD in the management of acute cerebral ischemia.

This research explored the neuroprotective effects of BTD using the TCM approach of replenishing vital energy, promoting blood circulation, and unblocking meridians. Buyang Huanwu Decoction, a well-established herbal prescription, has been extensively investigated, and contemporary research has demonstrated its capacity to enhance neurological recovery and modulate critical signaling cascades, including AKT1, JAK/STAT, and TLR4/NF-κB/NLRP3 pathways (24-26). Using BHD as a positive control, this study validated that BTD has comparable therapeutic efficacy. The comparison between BTD and BHD was descriptive rather than statistical; BTD showed a numerically stronger trend, but no claim of statistical superiority is made.

Furthermore, modern meridian-unblocking drugs, such as Tongxinluo, have robust evidence-based data supporting their clinical efficacy (27, 28). This study investigated the cellular and molecular mechanisms by which BTD inhibits neuronal inflammatory pyroptosis, thereby providing a more refined scientific explanation of meridian-unblocking efficacy at the level of programmed cell death pathways. In addition, although existing research has confirmed that multiple TCM formulations can suppress pyroptosis through modulation of the NLRP3 pathway (29, 30), this study used a parallel-control design with a classical formula and integrated multiple experimental approaches, including transmission electron microscopy and protein expression analysis. This design provided a more intuitive and comprehensive mechanistic chain of evidence.

Notably, the overall efficacy of BTD is likely attributable to its systemic protective effects. Cerebral ischemic injury is not an isolated event; rather, it is a dynamic interactive process involving neurons, astrocytes, microglia, and vascular endothelial cells (31). This study observed that BTD not only improved neuronal structure but also promoted restoration of cerebral blood flow, suggesting both neuroprotective and vasoregulatory functions. Contemporary scientific attention has shifted toward the gut–brain connection in stroke pathogenesis, whereby gastrointestinal microbial imbalance can intensify peripheral and central inflammatory responses and ultimately influence clinical outcomes (32). Notably, key botanical constituents of BTD, including *Astragalus membranaceus* and *Salvia miltiorrhiza*, have demonstrated efficacy in regulating microbial communities and enhancing intestinal barrier integrity (33, 34). These findings suggest that the anti-inflammatory and neuroprotective properties of BTD may be mediated, at least in part, through preservation of gut homeostasis. This potential mechanism warrants further investigation.

The current study has several limitations. First, although batch consistency was verified through assessments of yield and concentration, the lack of detailed chemical profiling of active constituents remains a limitation for the reproducibility of this herbal formula. Metabolomic techniques such as UPLC-Q-TOF/MS can be used for further identification and analysis of BTD. Second, the study used a prophylactic-like dosing regimen starting 24 hours after reperfusion; further studies should include postreperfusion interventions that more closely simulate clinical treatment scenarios. Third, neurological function was assessed only at baseline and at the endpoint. The lack of serial behavioral evaluations during the intervening period limits the temporal resolution of functional recovery data. Future studies should incorporate more frequent assessments to better characterize the dynamic course of neurological improvement after BTD treatment. Fourth, this investigation did not thoroughly examine the effects of BTD on vascular regeneration and metabolic adaptation in ischemic cerebral regions. These pathological processes are closely intertwined with pyroptosis and inflammatory responses and therefore warrant integrated investigation in future research to fully elucidate the comprehensive neuroprotective mechanisms of BTD. Finally, because the present study did not include NLRP3/Caspase-1/GSDMD pathway-specific inhibition or rescue experiments, the mechanistic conclusions remain associative rather than causal. Future studies using these approaches are needed to confirm causality.

### 5.1. Conclusions

In summary, this study provides suggestive evidence that BTD is associated with mitigation of AIS in conjunction with suppression of pyroptosis mediated by the NLRP3/Caspase-1/GSDMD pathway. Its neuroprotective mechanism is not confined to a single pathway but is likely embedded within a network involving regulation of hypoxic stress, maintenance of neurovascular function, and systemic immune modulation. These results provide theoretical and experimental foundations for advancing the application of BTD in animal models of AIS therapy.

ijpr-25-1-171161-s001.pdf

## Data Availability

The dataset presented in the study is available on request from the corresponding author during submission or after publication. The data are not publicly available due to the ongoing nature of the research and the need to protect unpublished data.
