# Supplementary material for: Buyang Tongluo Decoction Mitigates Acute Ischemic Stroke by Suppressing NLRP3/Caspase-1/GSDMD Pathway-Induced Pyroptotic Cell Death
Source: Iran J Pharm Res. 2026 Jun 9;25(1):e171161. doi: 10.5812/ijpr-171161 (PMC13769052; doi:10.5812/ijpr-171161)
Supplement: Supplementary file 1 [file ijpr-25-1-171161-s001.pdf]

Table S1. Composition of Buyang Huanwu Decoction (BHD) and Buyang Tongluo Decoction (BTD)

Formulation	Latin name	English name	Chinese name	Medicinal part	Weight (g)
Buyang Huanwu decoction (BHD)	*Astragalus membranaceus* (Fisch.) Bunge.	Astragalus Root	Huangqi	Root	120.0
	Angelica sinensis (Oliv.) Diels.	Chinese Angelica	Danggui	Root	4.5
	Paeonia veitchii Lynch.	Red Peony Root	Chishao	Root	4.5
	Pheretima aspergillum (E.Perrier).	Earthworm (dried)	Dilong	Female desiccant	3.0
	Ligusticum chuanxiong Hort.	Sichuan Lovage Rhizome	Chuanxio ng	Root	3.0
	Semen persicae (L.) Batsch.	Peach seed	Taoren	Seed	3.0
	Carthamus tinctorius L.	Safflower	Honghua	Flower	3.0
Buyang Tongluo Decoction (BTD)	*Astragalus membranaceus* (Fisch.) Bunge.	Astragalus Root	Huangqi	Root	60.0
	Angelica sinensis (Oliv.) Diels.	Chinese Angelica	Danggui	Root	6.0
	Paeonia veitchii Lynch.	Red Peony Root	Chishao	Root	6.0
	Pheretima aspergillum (E.Perrier).	Earthworm (dried)	Dilong	Female desiccant	5.0
	Ligusticum chuanxiong Hort.	Sichuan Lovage Rhizome	Chuanxio ng	Root	3.0
	Semen persicae (L.) Batsch.	Peach seed	Taoren	Seed	5.0
	Carthamus tinctorius L.	Safflower	Honghua	Flower	3.0
	Saposhnikovia divaricata (Turcz.) Schischk.	Saposhnikovia Root	Fangfeng	Root	5.0
	Ephedra sinica Stapf	Ephedra	Mahuang	Aerial part	3.0
	Cinnamomum cassia (L.) J.Presl	Cassia Twig	Guizhi	Twig	3.0
	Paeonia lactiflora Pall.	Peony Root	Shaoyao	Root	10.0
	Glycyrrhiza uralensis Fisch.	Liquorice Root	Gancao	Root & Rhizom	6.0
	Prunus armeniaca L.	Apricot Seed	Xinren	Seed	9.0
	Pueraria lobata (Willd.) Ohwi	Kudzu Root	Gegen	Root	25.0
	Notopterygium incisum T.T.Ching ex H.T.Chang	Notopterygium Root	Qianghuo	Root & Rhizom	3.0

Table S2 Summary of initial animal numbers, exclusions with reasons, replacements, and final analyzed sample sizes across different experimental endpoints.

Endpoint	Group	Initial N	Excluded (reason)	Replaced	Final N analyzed
Neurological score (24h & 14d)	Sham	14	0	0	12
	Model	14	2 (subarachnoid hemorrhage)	2	12
	BHD	14	0	0	12
	BTD	14	0	0	12
CBF (laser speckle)	Each group	–	0	–	5 (random subsample)
Infarct volume (TTC)	Each group	–	0	–	3 (random subsample)
Western blot, ELISA	Each group	–	0	–	3 (random subsample)
